# Coordinated Management of COVID-19 Response: Lessons From Whole-of-Society and Whole-of-Health Strategies in Wuhan, China

**DOI:** 10.3389/fpubh.2021.664214

**Published:** 2021-08-03

**Authors:** Shanquan Chen, Pan Zhang, Yun Zhang, Hong Fung, Yong Han, Chi Kin Law, Zhiqiang Li

**Affiliations:** ^1^School of Clinical Medicine, University of Cambridge, Cambridge, United Kingdom; ^2^Institute of Hospital Management, ZhongNan Hospital of Wuhan University, Wuhan, China; ^3^Program in Public Health, Renaissance School of Medicine, Stony Brook University, Stony Brook, NY, United States; ^4^Executive Director and Chief Executive Officer of CUHK Medical Centre (CUMC), Hong Kong, China; ^5^Jockey Club School of Public Health and Primary Care, Chinese University of Hong Kong, Hong Kong, China; ^6^Institute of Hospital Management, ZhongNan Hospital of Wuhan University, Wuhan, China; ^7^NHMRC Clinical Trials Centre, Faculty of Medicine and Health, University of Sydney, Camperdown, NSW, Australia; ^8^Vice-President of Dongxihu Fangcang Hospital, Wuhan, China; ^9^Vice-President of Zhongnan Hospital, Wuhan University, Wuhan, China

**Keywords:** COVID-19, epidemic, coordinated management, Wuhan, document analysis

## Abstract

**Background:** The outbreak of novel coronavirus disease 2019 (COVID-19) has been challenging globally following the scarcity of medical resources after a surge in demand. As the pandemic continues, the question remains on how to accomplish more with the existing resources and improve the efficiency of existing health care delivery systems worldwide. In this study, we reviewed the experience from Wuhan - the first city to experience a COVID-19 outbreak – that has presently shown evidence for efficient and effective local control of the epidemic.

**Material and Methods:** We performed a retrospective qualitative study based on the document analysis of COVID-19-related materials and interviews with first-line people in Wuhan.

**Results:** We extracted two themes (the evolution of Wuhan's prevention and control strategies on COVID-19 and corresponding effectiveness) and four sub-themes (routine prevention and control period, exploration period of targeted prevention and control strategies, mature period of prevention and control strategies, and recovery period). How Wuhan combatted COVID-19 through multi-tiered and multi-sectoral collaboration, overcoming its fragmented, hospital-centered, and treatment-dominated healthcare system, was illustrated and summarized.

**Conclusion:** Four lessons for COVID-19 prevention and control were summarized: (a) Engage the communities and primary care not only in supporting but also in screening and controlling, and retain community and primary care as among the first line of COVID-19 defense; (b) Extend and stratify the existing health care delivery system; (c) Integrate person-centered integrated care into the whole coordination; and (d) Delink the revenue relationship between doctors and patients and safeguard the free-will of physicians when treating patients.

## Introduction

The novel coronavirus disease 2019 (COVID-19) was declared a pandemic by the World Health Organization (WHO) on March 11th, 2020 (1). In response to the outbreak, a global challenge ensued from a shortage of medical resources as demands spiked. Worldwide, different responses were tested in the fight against COVID-19, such as the Fangcang hospital in Wuhan (2), the hospital ship in the United States (3), the COVID-19 track and trace app in Singapore, Australia, the UK, and Africa (4–6), and the rapid testing kit and hands-free hand sanitisers in Africa (6). These responses have solved problems to some extent, but remain highly inadequate from the health care delivery system perspective. Critically, the WHO outlined in its guidelines that systemic and cooperated strategies are key in managing COVID-19 response (7–9). However, how to incorporate this in practice, that is, into existing health care delivery systems, is still a learning process globally.

As the first city affected by COVID-19, Wuhan fought against the outbreak with little insight, but controlled the then epidemic, over a short duration, with impressively limited rebound (10, 11). Wuhan's experience, especially in pioneering the use of non-pharmaceutical interventions (NPIs) (12) and erecting Fangcang shelter hospitals (2), created a framework for COVID-19 response globally. Although some studies have summarized and disseminated the experience of Wuhan (2, 13), no study has thus far reviewed Wuhan's responses from a “system” perspective, with the introduction on how multi-tiered and multi-sectoral collaboration was performed locally and, importantly, how existing health fragmentation in Wuhan was overcome in response to COVID-19.

In this study, we bridged the knowledge gap and aimed to give more practical support to regions or countries suffering from COVID-19 and for future epidemics.

## Materials and Methods

### Study Setting

Wuhan is the capital city of Hubei Province and a major transportation hub in central China with a population of around 11.2 million (about 14% of them are aged 65 or over). By the end of 2018, Wuhan had 6,340 health institutions (including 398 hospitals), 10.8 beds per 1,000 citizens, and 4.5 physicians per 1,000 citizens (14). However, like the overall situation in China, the health care delivery system of Wuhan is fragmented, hospital-centred, and treatment-dominated, with little effective collaboration among institutions in different tiers(detail in supplementary (**Box 1**) (15, 16). Additionally, the lack of a mandatory primary care system via general practitioners and quality medical resources mainly occupied by hospitals allowed residents of Wuhan to have unrestrained access to second or tertiary health services. This further exacerbated fragmentation and lowers the efficiency of the health care delivery system, particularly where minor health concerns are experienced. In such cases, where primary care services would perhaps suffice, specialized health care is being unduly overwhelmed and exploited.

### Study Design and Data Collection

We performed a retrospective qualitative study based on the document analysis of COVID-19-related materials and interviews with first-line people in Wuhan. The materials included official policies, technical guidelines, and reports related to COVID-19. The materials were retrieved using terms related to COVID-19 (Chinese words included “新冠”, “新型冠状”, and “肺炎”) from the official webpage at the national level, city level, and community level, published during December 01, 2019 and March 20, 2020. Data were extracted by two investigators (SC and PZ) using a standardized form including the following domains: publication date, objectives/challenges, participants-involved, and the functions and responsibilities of the participants. All retrieved materials were screened independently by two investigators (SC and PZ). We only included materials related to COVID-19. Discrepancies in the inclusion or exclusion of materials during screening were discussed with a third reviewer (YH) until consensus was achieved.

Authors (PZ, YH, and ZL) have been in the front-line of the response since early 2020. Their affiliation is the designated hospital for COVID-19; therefore their first-hand experience was also used in the study. Specifically, with regard to information on designated hospitals, including patient screening, transfer, and treatment procedures, we interviewed hospital leaders and other front-line staff within departments in charge of epidemic prevention and control, including the department of infectious diseases, emergency department, fever clinic, and department of hospital external liaison. For information about Fangcang hospitals, besides the information from author (ZL), who was the deputy director of one of the Fangcang hospitals, we also interviewed the leaders of the national rescue team in Hubei and the leaders of the districts, who were involved in the operation of the Fangcang hospital and the patient transfer process. Regarding the community and isolation points, we interviewed the staff from affiliated community centers and isolation points; information about the deployment of the command center of prevention and control is mainly based on interviews with staff who were second to the command center, including resource allocation, department coordination, and information release at various stages.

The measure of effectiveness involves at least two concepts, namely cost and outcome. There is an article that estimates the corresponding costs (17), but because Wuhan is the first city to face the COVID-19 outbreak and many measures are generated and improved in the process of exploration, so the corresponding costs are much higher than those of truly effective measures. Also, because China uses the power of the whole country to control the spread of the outbreak in the shortest possible time at all costs, effectiveness based on cost and outcome does not have a particularly significant reference value. Here, we just used the outcome to reflect the effectiveness. Similar handling can be found in a publication (2). The outcome was measured by confirmed cases, death cases, and recovery cases of COVID-19 in Wuhan, and data was collected from the official webpage (18).

### Data Analysis

Initial extracted data were reviewed by all authors, and two themes and four sub-themes were formulated. The two themes are the evolution of Wuhan's prevention and control strategies on COVID-19 and corresponding effectiveness. The four sub-themes are routine prevention and control period, exploration period of targeted prevention and control strategies, mature period of prevention and control strategies, and recovery period. Data were re-coded into a matrix (Table 1 is its compact edition).

**Table 1:** Participants and corresponding strategies of COVID-19 prevention and control in Wuhan^†^^‡^.

**Participants**	**Phase 1: routine prevention and control period (12/27/2019-01/21/2020, 26 days)**		**Phase 2: exploration period of targeted prevention and control strategies (01/22/2020-02/04/2020, 14 days)**		**Phase 3: mature period of prevention and control strategies (02/05/2020-03/10/2020, 35 days)**	**Phase 4: recovery period (03/11/2020-present)**
	**12/27/2019-01/15/2020**	**01/16/2020-01/21/2020**	**01/22/2020-02/01/2020**	**02/02/2020-02/04/2020**		
**Health Administration, and Centers for Disease Control and Prevention**	1. Control the outbreak point (a seafood market in Wuhan). 2. Controlling public transportation occasions such as airports, stations, and wharves. 3. Trace and explore the source of disease. 4. Clear that the pathogen of unknown pneumonia is a new type of coronavirus.	1. Develop initial diagnosis and treatment and prevention and control plans for COVID-19.	1. Lockdown. 2. Designated, expansion, and building dedicated hospitals for COVID-19. 3. Extend pathogen detection technology from national-level units to provincial-level units, and then to city-level units.	1. Clarified four types of key groups that need to be monitored (including fever patients and their close contacts, suspected COVID-19 patients, confirmed COVID-19 patients), and corresponding management methods. 2. Initially clarified the functional role of the community in COVID-19 prevention and control.	1. Extend technical support for COVID-19 prevention and control to the community level. 2. Clarified the functional role of designated hospitals such as public hospitals in COVID-19 prevention and control. 3. Ensure the safe and efficient operation of the entire system and responses to emergencies, by deploying material and human resources.	1. Primary focus on the community to prevent the COVID-19 from rebounding. According to the number of newly confirmed cases divided the community into low, medium, and high-risk area. 2. City re-open.
**Public or designated hospitals**	X	1. Screen suspicious patients who go to the hospital.	1. Screen suspicious patients who go to the hospital.	1. Screen and treat suspicious patients who go to the hospital.	1. Only provide services for moderate or severe COVID-19 patients.	1. Provide nucleic acid test. 2. Provide treatment services to confirmed COVID-19 patients.
**Fangcang shelter hospitals**	X	X	X	X	1. Come into service. 2. Clarified the functional role of Fangcang Hospital, which only provides treatment services to mild confirmed COVID-19 patients.	1. Gradually closed.
**Independent quarantine center**	X	X	1. Started to form, dedicated to isolating suspicious patients.	1. Initially clarified the functional role of the quarantine point. The quarantine points are divided into two categories: “admission” and “to be discharged”. The former only accepts patients with suspected fever and confirmed positive patients, and the latter only accepts patients who are discharged from the hospital after a negative nucleic acid test.	1. Accepts suspected cases from the community and conducts a decisive nucleic acid test to determine the final flow of patients. 2. Accepts recovered cases from the hospital for the second nucleic acid test to avoid recurrence before the patient returns to the community.	1. Gradually closed.
**Community and primary care**	X	X	1. Began to intervene, mainly to check body temperature.	1. The community began to classify and manage the four types of patients. However, due to the lack of technology, the main focus of the community is on checking the numbers of confirmed and suspected patients, and the prevention and control functions at the community level have not been fully utilized. Community-level personnel flow control has not been achieved.	1. Screen the population and classifies the four types of population via common examinations, likes blood routine and chest imaging. 2. Provides health education on COVID-19 prevention.	1. Medium to high-risk community: continue to maintain the original work, including the screening of suspected cases and the control of crowd activities. 2. Low-risk community: Screen external people to prevent the importation of external COVID-19 cases.
**Key features**	1. Lack of evidence for COVID-19. 2. Routine prevention and control, but lacks a reasonable targeted strategy. 3. The screening process is cumbersome and time-consuming. The first-line units are mainly hospitals.		1. Identify the population that needs to be monitored. 2. The main body of COVID-19 prevention and control has gradually transitioned from simplification to diversification, and the functional role of each main body has been clarified. A multi-agent cooperation model has taken shape.		1. Final multi-agent cooperation model.	1. Social order and medical order are gradually returning to normal. 2. Only keep the prevention and control at the community level and the hospital level, especially at the community level.

† *The evolution of Wuhan's prevention and control strategies on COVID-19 was divided into four periods based on three milestone: the date of determining that COVID-19 can be transmitted from person to person (January 22, 2020); putting the Fangcang hospital into use and providing technical support for community and primary care (February 05, 2020); and the day that the daily number of new confirmed COVID-19 cases has fallen below ten (March 11, 2020)*.

‡ *X in table cell means not involved*.

All coding was done in Excel. How Wuhan combatted COVID-19 through multi-tiered and multi-sectoral collaboration, overcoming its disadvantaged fragmented, hospital-centered, and treatment-dominated healthcare system, was illustrated and summarized.

Demonstrating effectiveness for each response using a systems approach is challenging in the case of Wuhan as changes in one part (for example, primary health centers) altered other parts of the system (like hospitals) in ways that are difficult to measure. In addition, measures in Wuhan were implemented over short intervals and led to high overlap, making the separation of effectiveness for each response even more complex. Therefore, the judgment of effectiveness was mainly based on the subjective account of front-line personnel: whether relevant measures are important to control the spread of the virus.

## Results

At the early stage of the COVID-19 outbreak, Wuhan's health care delivery system was heavily challenged. The health needs and panic incurred by COVID-19 overloaded the hospitals in Wuhan, and resulted in the serious outcome of limited accessibility and overcrowding. These outcomes demonstrated the weaknesses of Wuhan's health care delivery system when facing the needs of COVID-19 prevention and control.

### The Evolution of Wuhan's Prevention and Control Strategies on COVID-19

**Figure 1:**
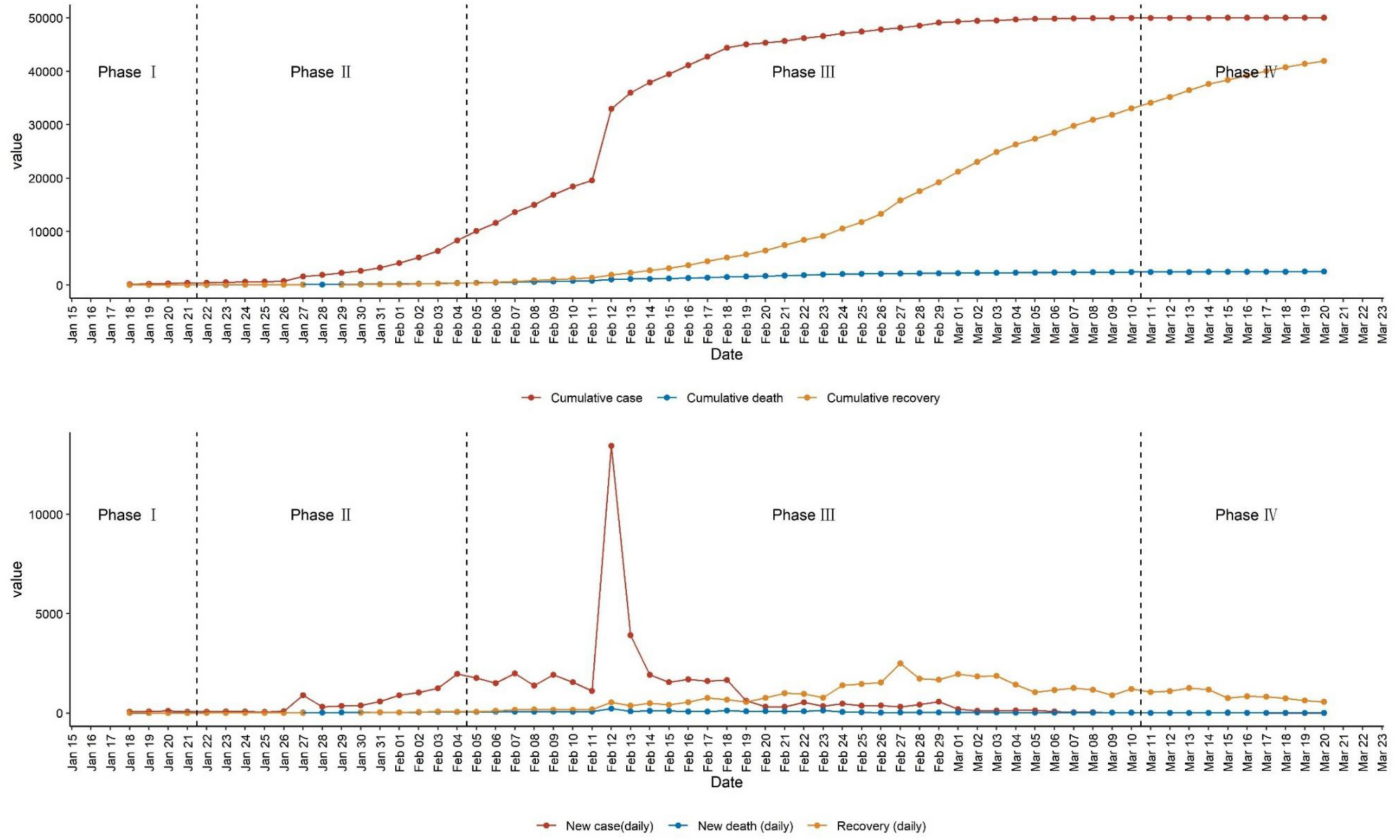
Confirmed cases, death cases, and recovery cases of COVID-19 in Wuhan. This figure shows the results for or from confirmed cases. Before March 10, 2020, suspected cases are determined based on both clinical manifestations (two of following three manifestations: 1, Fever; 2, has imaging features of pneumonia; and 3, the total number of white blood cells is normal or decreased, or the lymphocyte count is decreased) and contact history (one of following three histories within 14 days before the onset of illness: 1, Have travel history or residence history in areas where the COVID-19 case continues to spread; 2, Contact with patients with fever or respiratory symptoms from areas where the COVID-19 case continues to spread; 3, There is a cluster of disease or an epidemiological association with the new coronavirus infection). Suspected cases are confirmed cases with one of the following two pieces of evidence: 1. Respiratory tract specimens or blood specimens' real-time fluorescent RT-PCR detection of new coronavirus nucleic acid positive; 2. Respiratory tract specimens or blood specimens' virus gene sequencing, and known new coronavirus Virus highly homologous. After March 10, 2020, the corresponding standards have become stricter. Suspected cases are determined only based on clinical manifestations (one of the following two manifestations: 1, Fever; and 2, the total number of white blood cells is normal or decreased, or the lymphocyte count is decreased).

There were three milestone for Wuhan's COVID-19 crisis: (i) the date of determining that COVID-19 can be transmitted from person to person (January 22, 2020); (ii) putting the Fangcang hospital into use and providing technical support for community and primary care (February 05, 2020); and (iii) the day that the daily number of new confirmed COVID-19 cases fell below ten (March 11, 2020). Accordingly, we divided the evolution of Wuhan's prevention and control strategies on COVID-19 into four periods. Table 1 summarizes the main participants in each period and their corresponding prevention and control strategies. Figure 1 shows the COVID-19 cases (including confirmed, death, and recovered) in Wuhan in each period.

**Period I**, ***routine prevention and control period***(Figure 1, from December 27, 2019, to January 21, 2020, 26 days), featured by the formidable COVID outbreak in lack of specific evidence on diagnosis, treatment, prevention, and control strategy.

During this period, COVID-19 was seen as an atypical pneumonia with an unknown pathogen. Workers of disease prevention and control were mainly public health administrative departments and hospitals. After the pathogen was identified as a novel coronavirus, the prevention and control strategies in this period were made based on conventional experience (not targeted) and with the hospital as the primary first-line units, following lack of specialized knowledge.

**Period II**, ***exploration period of targeted prevention and control strategies***(Figure 1, from January 22, 2020 to February 04, 2020, 14 days), featured by a combination of chaos and exploration.

From January 22, 2020, following the official announcement that COVID-19 is a human-to-human infection disease, Wuhan entered the road of exploring targeted prevention and control strategies. China has experienced many acute infectious disease emergencies, including Severe Acute Respiratory Syndrome (SARS) in 2003 (19), the H1N1 flu epidemic in 2009 (20), the H7N9 avian flu epidemic in 2013 (21), and Middle East Respiratory Syndrome (MERS) in 2015 (22). Drawing from these experiences, China has been gradually improving its health-emergency-related surveillance, preparedness, and response capacities. At the initial point of this period, a top-down temporary directive team was organized, led by the provincial governor and composed of the provincial-city-community level of heads of health, public security, civil affairs, finance, human resources, transportation, economy, information, and news.

The series of anti-epidemic measures implemented during this period were derived from past anti-epidemic experiences, such as social distancing and the establishment of hospitals. This perhaps explains why Wuhan could respond quickly and decisively from when COVID-19 was identified as a human-to-human infection disease. Not all empirical measures applied to this new type of virus, but the determination to control the epidemic at all costs determined the rapid response of Wuhan's epidemic prevention and control, and laid the foundation to identify effective protective measures e.g., social distancing and movement control order, later recommended by the WHO. To further enhance the ability of rapid learning and adaptation, feedback from first-line anti-epidemic measures and related issues were collected and aggregated to the central control team. For instance, to fully implement body temperature screening in various places and strengthen the monitoring of fever clinics in medical institutions, requisite 2-hour direct network reporting, 12-hour feedback of test results, and complete on-site epidemiological investigation within 24 hours were demanded.

The most well-known measure taken by Wuhan is the city-wide lockdown from January 23, 2020. The lockdown of Wuhan confined the outflow of possible contaminated populations and constrained the spread of COVID-19. But the sudden closure also brought a great negative impact on the normal function of the whole city and instituted panic among the citizens. There were three reasons for the serious overcrowding and cross-infections within the hospitals: the lack of sufficient buffer time meant the prevention and control strategies within Wuhan had not been completely synchronized with the closure; Wuhan itself had a fragmented, hospital-centred, and treatment-dominated health care delivery system; and (c) a large volume of individuals (both COVID-19 patients and non-COVID-19 patients) -presented at hospitals with to an irrational fear of virus infection (COVID-19 or seasonal flu). As a result, initially, the city-wide lockdown not only failed to well-control the spread of the COVID-19 within Wuhan, but instead impeded COVID-19 patients getting timely screening and health services. The transient rise in the number of cases between Jan 26 and Jan 29 (**Figure 1, phase II**) could be partially explained by this failure. Furthermore, the health services for patients with non-respiratory diseases, like women in labour, were also influenced (23).

The negative results above forced Wuhan to adjust its hospital-centred strategies. To meet the needs beyond the COVID-19 patients and to reduce the nosocomial infection, Wuhan re-designed the roles of hospitals in the system response (e.g., some hospitals were designated as infectious disease hospitals admitting COVID-19 patients, while some were designated for treating patients with other urgent or non-urgent conditions), and included community and primary care as the first line to prevent and control COVID-19. In addition, the construction of independent quarantine centres for the infected cases, carried out almost at the same time as the city-wide lockdown, was gradually completed. Up to February 01, 2020, a multi-tiered and multi-agent prevention and control strategy has begun to take shape. However, cooperation between agents was still absent at this time, because the functional role of each agent had not yet been confirmed. For instance, community and primary care at this time mainly helped to measure body temperatures, and were far from fulfilling their first-line role.

According to the available evidence at this stage, on February 02, 2020, Wuhan clarified four special groups of the population who needed to be screened and monitored for COVID-19, namely fever patients, their close contacts, suspected COVID-19 patients, and confirmed COVID-19 patients. Meanwhile, the functional role of the community and primary care and independent quarantine center was initially determined. Community and primary care were positioned to help classify and manage the above four types of population. Independent quarantine centers were divided into two categories (“admission” and “to be discharged”) to reduce the cross-infection within and the pressure on the hospital. The “admission” quarantine center only accepted patients with suspected fever patients or confirmed positive patients, and the “to be discharged” quarantine center only accepted patients who were discharged from the hospital after a negative nucleic acid test. By February 02, 2020, a multi-tiered and multi-agent cooperation prevention and control strategy had begun to take shape.

To screen the four key groups of people for COVID-19, Wuhan began to screen the general population for the first time from February 03, 2020. This time, screening encompassed the grand mobilization of the whole city, but still failed because screening (prevention) and control were not fully coordinated. Due to the lack of technical support, the community and primary care still passively fought COVID-19: the main energy of the community and primary care had been spent on checking the numbers of suspected and confirmed patients, and failed to take control of community residents in points of entry, resulting in the movement of people still being common. This failure was also reflected in the comparison between the newly confirmed cases during this time screen (after February 03, 2020) and those in phase 3 (after February 08, 2020, illustrated below) (Figure 1), which was seminal to the COVID-19 defence success in Wuhan. Nevertheless, although a kind of negative result, it laid the foundation for the evolution of the multi-agent cooperation model.

In summary, this period was a combination of chaos and exploration, and Wuhan showed a quick response capacity. During this period, four types of the population were identified, the main body of COVID-19 prevention and control gradually transitioned from simplification to diversification, and the functional role of each agent was initially clarified.

**Period III**, ***mature period of prevention and control strategies***(Figure 1, from February 05, 2020 to March 10, 2020, 35 days), featured by a well operated multi-tiered and multi-agent cooperation.

**Figure 2:**
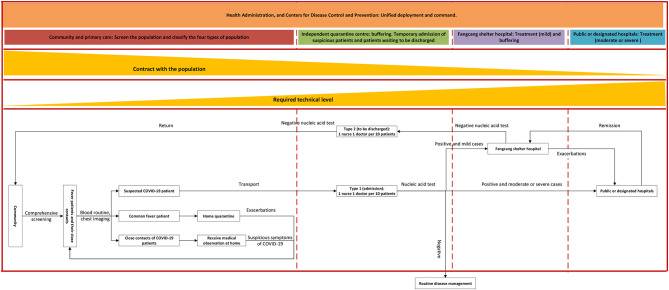
Multi-agent cooperation model of COVID-19 preventing and controlling in Wuhan.

From February 05, 2020, Fangcang shelter hospitals had begun to be put into service, and on February 06, 2020, infection control specialists were sent to community and primary care clinics to provide technical support, marking that Wuhan's COVID-19 prevention and control model had entered a mature period. Technical support encompassed vetting environmental characteristics of the community and the composition of residents, developing targeted prevention and control measures, including how to screen, how to educate and raise awareness, how to transfer confirmed patients, how to deal with domestic waste, how to disinfect the community, and how to provide psychological assistance. Figure 2 visualizes the final multi-tiered and multi-agent cooperation model in Wuhan.

In this final model, different from the regular health care delivery system in Wuhan, all referrals of patients now had to go through primary care physicians, and the advanced hospital had the right to refuse patients' over-specialized/advanced needs if they bypassed primary care. ***Community and primary care***services helped to manage and screen the population and classify the four types of the population via physical examinations, routine blood screening, and chest imaging, as well as the provision of health education on COVID-19 prevention. ***Independent quarantine centers*** functioned as a buffer between screening and treatment. These centers not only accepted suspected cases from the community and conducted confirmatory nucleic acid tests to determine the final flow of patients, but also accepted recovered cases from the hospital for the second nucleic acid test to ensure a non-infectious state, before the patient returned to the community. ***Fangcang shelter hospitals***provided treatment services to mild confirmed COVID-19 patients and also functioned as a buffer between independent quarantine centers and tertiary hospitals to alleviate the burden on tertiary hospitals, by accepting the mild COVID-19 patients from the quarantine center and the COVID-19 patients from tertiary hospitals whose symptoms had turned from moderate or severe to mild. ***Public or designated hospitals***usually are tertiary hospitals and specifically aim to provide services for moderate or severe COVID-19 patients. ***Health*** ***administration, and Centers for Disease Control (CDC) and Prevention***worked as the central command center, ensuring the safe and efficient operation of the entire system, and promptly responded to emergencies by deploying material and human resources.

The cooperation of Wuhan's model in practice combines the integration of both **horizontal** and **vertical cooperation**. Figure 2 is a direct demonstration of how different agents in Wuhan collaborate vertically, while horizontal cooperation is mainly reflected by cooperation between each agent and command center, as well as the work within the community and primary care. Through the big data platform, the command center contributed to a whole-of-health and whole-of-society system by integrating the data of hospitals, CDC, community and primary care, medical insurance, polices, civil affairs, communications, and other related departments, which not only facilitates the screening of the population, but also ensures that no contaminated cases are omitted. In addition, the announcement of free COVID-19-related services thoroughly disconnected the long-term criticized revenue-linkage between doctors and patients, and increased people's confidence and compliance with the official anti-COVID-19 decisions (e.g. mask-wearing and maintaining social-distancing).

The horizontal cooperation within each community was mainly done by a team with at least five members, consisting of community medical staff, community officials, community volunteers, and community police. This team together were in charge of about 300 families (800 populations) and responsible for the screening, health education, mental health support, daily living support for special populations such as the elderly living alone and disabled people, and the control of unnecessary mobility of residents. It is worth emphasizing that it is the work at the level of community and primary care that greatly promoted the practical implementation of prevention and control measures. Unlike the initial response to COVID-19, at this stage, people had a certain understanding of this disease (through official media and internal education in the community) and gradually realized the important role of individual-level cooperation in the overall anti-COVID-19 progress. Under the encouragement of the community team, people engaged more in online activities to reduce physical contact with each other.

From February 08, 2020, Wuhan city began to screen the entire population for the second time to distinguish the four key groups of people, and more than 10 thousand people were screened (Figure 1). From February 16, 2020, to February 19, 2020, Wuhan conducted the third all-population screening to ensure that no contaminated population were unidentified. In hindsight, it was the second screening that predicted the arrival of Wuhan's success in fighting the COVID-19, with the third screening consolidated Wuhan's anti-COVID-19 results.

**Period IV**, ***recovery period***(Figure 1, from March 11, 2020, to present), featured by community and primary care-led COVID-19 campaign.

From March 11, 2020, the daily number of new confirmed COVID-19 cases had fallen below ten (Figure 1), marking that Wuhan's COVID-19 prevention and control strategy had entered a recovery period. During this period, social order and medical order were gradually returning to normal, for instance, independent quarantine centers and Fangcang shelter hospitals were shutting down. However, the prevention and control at the community level had not been loosened but instead been adjusted according to actual needs. The communities were divided into low, medium, and high-risk areas, based on the number of newly confirmed cases. Only a community that satisfied two conditions could be recognized as a COVID-19-free community: a) there were no new confirmed case of COVID-19 for more than 14 days and b) no new case of three types of personnel (suspected COVID-19, fever cases, and close contacts) within 14 days. Communities assessed at different levels of risks adopted different corresponding prevention and control strategies to prevent the rebound of COVID-19 (Table 1).

In addition, for various institutions and places, Wuhan had formulated corresponding strict prevention and control strategies. For instance, operation of entertainment and leisure venues were suspended until June 12 until the daily number of new confirmed COVID-19 cases had fallen below ten from March 11, 2020; in public places such as shopping malls, supermarkets, bookstores, and bazaars, temperature checks were required for entrance, and customers were urged to wear masks; hotels, restaurants and other catering establishments were required to extend table spacing in addition to controlling the number of diners. Furthermore, another two large-scale screenings were carried out to ensure that COVID-19 would not rebound. On April 14th, the Chinese CDC launched the COVID-19 seroepidemiological sample survey in Wuhan, and the sampled number reached 11 thousand. Later, on May 2nd, Wuhan required all communities to complete nucleic acid screening for all members of their jurisdiction within 10 days.

### Effectiveness

From the date when COVID-19 was confirmed as a human-to-human infection, to the date when the daily number of new confirmed COVID-19 cases reached more than ten thousand, and then to the date when the daily number of new confirmed COVID-19 cases had fallen below ten, Wuhan has experienced 49 days (**phase II and phase III**, Figure 1), including 14 days of exploration on the specific anti-COVID-19 strategies (**phase II**, Figure 1). These short periods demonstrate the rapid response of Wuhan to the epidemic and the efficacy of Wuhan's coordinated approach in fighting against COVID-19.

The only obvious pulse in panel 2 of Figure 1 predicted the arrival of Wuhan's success in fighting against the COVID-19. It indicates that Wuhan's coordinated model is not only strong in identifying suspicious COVID-19 cases, but also strong in accommodating and controlling the spread of virus borne by the patients. Up to 15 July 2020, the COVID-19 related mortality in Wuhan is 5.43% (11), lower than the average worldwide (24). Wuhan's low mortality indicates that the coordinated model matches the health needs of COVID-19 patients very well. Finally, Wuhan's coordinated approach has a property of thoroughness in fighting against COVID-19 as there is no rebound after COVID-19 was controlled (Figure 1).

## Discussion

Prevention and control of COVID-19 is a comprehensive task involving all fields of health and beyond, and requires an efficient multi-sectoral collaboration (7–9). WHO gave a comprehensive illustration of the cooperation strategies at the international level, but corresponding illustrations are absent or too ambiguous at the national or subnational level. The experience of Wuhan we introduced resonates with what WHO emphasized. More importantly, the experience we introduced here presents the dynamic changes of each participants' function, for instance, the role of community and primary care in the cooperated model changed in different stage. The dynamic function or contribution of each participant could be more practical. We believe the lessons learned from Wuhan are useful to other settings, especially in resource-limited settings.

Here are four lessons for the future drawn from Wuhan's experience:

First, engage the community and primary care services not only on supporting (equivalent to the **phase II** of the Wuhan experience) but also on screening and controlling (equivalent to the **phase III** of the Wuhan experience), and retain it as the first line of anti-COVID-19 (equivalent to the **phase III** and **Period IV**, of the Wuhan experience). Although COVID-19 has brought a great burden to the health care delivery system, most of the population who entered into the health care delivery system are suspected cases or mildly ill patients. In other words, most of them do not require advanced services. As shown in Figure 2, community and primary care are the health institutions that have the broadest contact with the general public, this property determined that community and primary care are the most appropriate unit to “scale-up case management, and conduct individual quarantine of cases, scale-up contact tracing and quarantine of contacts,” emphasized by the WHO (8). Wuhan's experience emphasized that if offered necessary technical support to community and primary care, this would greatly alleviate pressure on the health care delivery system and meet people's health needs at the same time (equivalent to the **phase III** of the Wuhan experience). The same effect was also verified by the experience in the UK, Brazil, Pakistan, and Ethiopia (25). However, although the proportion of countries and territories that have a community engagement plan rose from 19 to 85% (7), the function of community and primary care in some countries like Sudan (26) were still not fully used, as here community and primary care are still designated to support people (equivalent to the **phase II** of the Wuhan experience).

A study (27) compared the anti-COVID-19 measures used by nine regions or countries in the Asia Pacific Region and Europe, which reported the contribution from community and primary care. Community and primary care of these regions or countries quickly detected suspected cases, and achieved better outcomes in fighting COVID-19 during the first wave, similar to Wuhan (27–29). Studies from Italy (30), Singapore (31), France (32), Australia (33), the US (34), and Vietnam (35) demonstrate how the community and primary care can help to halt the Covid-19 pandemic by facilitating the implementation of related measures and maintain adequate health services. However, it is unclear why these regions or countries had a rebound or second wave of COVID-19, unlike Wuhan. We speculate that this to some extent could be due to the lack of contribution from community and primary care, equivalent to the fourth stage of the Wuhan experience.

Second, extend and stratify the existing health care delivery system. From suspected patients to confirmed mild patients, confirmed moderate or severe patients, the professional and quality of required health care by each group are different. Stratified patient care (equivalent to the **phase III** of the Wuhan experience) could distribute the medical burden and to some extent prevent a shortage of resources like beds. The same effectiveness was verified by the experience in South Korea (36). There is no that community and primary care centers should take charge of the suspected patients and (tertiary) hospitals should take charge of confirmed moderate or severe patients, but there exists variance in the arrangement of confirmed mild patients. Quarantining the mild patients at home not only increases the burden on the community and primary care and the risk of internal infections between families, but also reduces their chance of receiving quality health care. However, quarantining the mild patients in the hospital will occupy advanced services and crowd out those who need it likes the practice in the first stage in the Wuhan experience. Wuhan's experience indicated that introducing a transitional station (like the independent quarantine centers and Fangcang hospital in Wuhan) by modifying facilities that were not originally used for medical purposes, for example, stadiums, could not only buffer the burden for both community and primary care and hospitals, but also meet patients' special need on quality health care (2).

Third, integrate person-centered integrated care (PCIC) into the system. The concept of person-centred care is related to the cooperation and fragmentation between institutions. Different from other diseases, failures at any link (the links from the detection of COVID-19 to the treatment to the rehabilitation) will cause the efforts of other links to be futile. The speciality of COVID-19 requires each participant to have a patient-centred awareness, and to track and record each patient. Besides, to overcome the lack of resources, the population must be weary of seeking over-specialized/advance medical services - unless health condition necessitates – akin to pre-COVID times (37). These are in line with the concept of PCIC, which has been emphasized by WHO (38) and believed to improve outcomes and experiences for people with multiple long-term and complex conditions (39). It can be said that it is the concept of PCIC that enables Wuhan to realize its collaborative multi-tiered and multi-agent model. Once people enter the path in Figure 2, the healthcare delivery system at that time must ensure that they complete the path according to the corresponding guidelines.

Fourth, delink the revenue relationship between doctors and patients and protect the safety of physicians to make the right decisions. The revenue-linkage between doctors and patients, as well as the deteriorating doctor-patient relationship, are two long-term criticized topics in China. Both results in the physicians in the hospital unwilling to refuse patients' over-specialized/advanced services in consideration of their safety or revenue. During the COVID-19 outbreak in Wuhan, the health administration disconnected the revenue relationship between doctors and patients by making COVID-19-related services free of charge, and ensured the safety of physicians to allocate resources under professional decisions. Both measures maximized the spirit of physicians' professionalism and enhanced the reasonable allocation of limited advanced health resources. These are another two necessary conditions for the multi-tiered and multi-agent model in Wuhan operating well, besides the above condition of PCIC.

Despite the success of the Wuhan COVID-19 response framework, there are a couple of notable weaknesses in Wuhan's approach. First, the existing cooperation model is mainly aimed at patients with or suspected of COVID-19. This has a strong absorption effect on related health resources, which could to a certain extent crowd out patients beyond COVID-19 from using health services. Theoretically, this problem could be more severe at the community and primary care level, as COVID-19-related work takes up almost all the energy of the relevant staff. However, a lack of related data disables us from verifying if the current model can fully guarantee the continuity of essential services for patients beyond COVID-19. Second, although the existing model considers people's mental health needs, a vital issue pointed out by studies (40–42), it is still at a basic level by offering humanistic care and lacks a systematic approach that is compatible with mental health services, for instance, regular assessments of the mental health of those under quarantine.

The strength of our study is that it is a health system-focused case study of Wuhan's response to the novel coronavirus outbreak. As the first jurisdiction to detect and respond to the outbreak, Wuhan used the experience of previous infectious diseases outbreaks and innovative public health judgement to respond to the rapidly changing epidemic. As aforementioned, Wuhan's response has many of the same features later recommended by WHO. Documenting how elements of the system in Wuhan responded will be valuable. Additionally, the framework of the four phases of the response and the two themes, evolution and adaptation of the response and its effectiveness, cover the dynamic changes of each elements' function, which could be instructive for other countries.

Our study suffers from the common limitation of case studies. The specific context of Wuhan (first site of widespread transmission, unique health system) prevents generalization of the findings to other settings. Wuhan had a well-staffed and -stocked primary care service that could be mobilized – even if it was fragmented and had poor links to the tertiary sector. Primary care services in many limited resource settings are neglected, and these places may not cope with screening suspect cases. Nevertheless, the experience of Wuhan is still useful, as our lessons emphasize offering necessary technical support to the workforce at the level of community and primary care to mobilize their contribution. Similar lessons could also be learned from the UK, where the temporary COVID-19 testing stations run under the control of trained staff could meet the initial needs of the population. The opening characteristics of residential areas in countries like the UK and US are different from the ones in Wuhan, where residential communities are often featured with limited access gates and are controlled by property management staff. Even so, our lesson of lockdown or open communities based on their risk is still useful, and similar measures can be seen in countries like the UK but at the city level instead of the community level.

Second, the outbreak in Wuhan, China is different from the ones in other countries now, which are usually featured by an outbreak at the national level. Wuhan's large population were served by many tertiary hospitals and specialist teams, and national wide workforces and medical materials were sent to Wuhan at that time. In addition, there are more tools available to control transmission than were available to Wuhan. However, of its characteristics of widespread transmission and quick control make Wuhan's experience still meaningful to future possible new and lacking related evidence outbreaks, to which quick responses are always needed.

Third, the findings of this study should be interpreted cautiously. As we mentioned in the data analysis, special conditions in Wuhan limited the application of a systematic approach to demonstrate the effectiveness for each response, and the judgment of effectiveness in this study is mainly based on the subjective account of front-line personnel. In addition, Wuhan's prevention and control measures, such as restricting the movement of people through blockade of communities, were quite rigorous in retrospect. Although these measures have indeed reduced the spread of the virus, it is difficult for us to give a conclusion as to what extent these measures contributed to the eventual outcome.

Fourth, though our health system-focused view gives a detailed explanation on Wuhan's rapid response to COVID-19, more contributions can be made by comparing the measures and outcomes among multi Asian countries, as other Asian countries were very concerned initially with the reports from China and rapidly enforced lockdown, social distancing, and track and trace activities to help contain the virus. The corresponding comparison could generate more generalized lessons.

## Conclusions

In this article, we reviewed lessons from Wuhan that can be learned from a system perspective, and illustrated how Wuhan combatted COVID-19 efficiently through multi-tiered and multi-sectoral collaboration by overcoming its fragmented, hospital-centered, and treatment-dominated healthcare delivery system. By the end of 2020, COVID-19 remained prevalent globally, with some regions or countries experiencing second waves. Even very robust health care delivery systems can be rapidly overwhelmed and compromised by the sudden outbreak. Therefore, this study could be of great significance for fragmented health delivery systems around the world, offering a concrete example of effective disease/infection/outbreak-control and management.

## Data Availability Statement

Publicly available datasets were analyzed in this study. This data can be found here: The data used in this study are available from webpage of Wuhan Municipal Health Commission (http://wjw.wuhan.gov.cn/ztzl_28/fk/tzgg/202005/t20200501_1213582.shtml).

## Author Contributions

All authors contributed substantially to the work reported: SC conceptualization, SC and PZ writing—original draft preparation, SC, HF, YZ, CL, and YH writing—review and editing, ZL supervision and project administration. All authors have read and agreed to the published version of the manuscript.

## Conflict of Interest

The authors declare that the research was conducted in the absence of any commercial or financial relationships that could be construed as a potential conflict of interest.

## Publisher's Note

All claims expressed in this article are solely those of the authors and do not necessarily represent those of their affiliated organizations, or those of the publisher, the editors and the reviewers. Any product that may be evaluated in this article, or claim that may be made by its manufacturer, is not guaranteed or endorsed by the publisher.
